# Relationship Between EAT‐10 Scores, Pharyngeal Residue Severity, and Penetration–Aspiration in Individuals With Idiopathic Parkinson's Disease: A FEES‐Based Correlation and Discriminative Performance Study

**DOI:** 10.1111/1460-6984.70335

**Published:** 2026-09-10

**Authors:** İbrahim Erensoy, Fatma Esen Aydınlı, Özlem Yaşar, Emel Tahir, Murat Polat, Murat Terzi

**Affiliations:** ^1^ Department of Speech and Language Therapy, Faculty of Health Sciences Ondokuz Mayıs University Samsun Turkey; ^2^ Department of Speech and Language Therapy, Faculty of Health Sciences Hacettepe University Ankara Turkey; ^3^ Department of Otolaryngology, School of Medicine Ondokuz Mayıs University Samsun Turkey; ^4^ Department of Neurology, School of Medicine Ondokuz Mayıs University Samsun Turkey

**Keywords:** dysphagia, EAT‐10, FEES, Idiopathic Parkinson's disease

## Abstract

**Background:**

Dysphagia is common in individuals with Idiopathic Parkinson's Disease (IwIPD) and affects both swallowing safety and efficiency. Pharyngeal residue and penetration–aspiration are key impairments associated with dysphagia in this population, yet their relationship across different bolus consistencies and volumes remains incompletely understood. The clinical role of patient‐reported outcome measures, such as the Eating Assessment Tool‐10 (EAT‐10), in relation to instrumental swallowing findings also requires further investigation. This study aimed (1) to examine the relationship between pharyngeal residue and penetration–aspiration across different bolus consistencies and volumes in IwIPD, and (2) to evaluate the discriminative performance of the EAT‐10 against FEES findings related to these swallowing impairments.

**Methods:**

Ninety‐seven IwIPD were included. Participants completed the EAT‐10 and underwent Flexible Endoscopic Evaluation of Swallowing (FEES). Ten swallowing trials were conducted using boluses of varying consistencies and volumes prepared according to the International Dysphagia Diet Standardisation Initiative (IDDSI). FEES recordings were independently rated by three clinicians using the Penetration–Aspiration Scale (PAS) and the Yale Pharyngeal Residue Severity Rating Scale (YPRSRS).

**Results:**

Larger bolus volumes were associated with higher residue severity and PAS scores across planned within‐consistency comparisons. Significant correlations were observed between PAS scores and both EAT‐10 and YPRSRS scores across consistencies and volumes (*ρ* = 0.56–0.91, *p* < 0.01). The EAT‐10 showed discriminative performance against FEES findings related to pharyngeal residue, penetration, and aspiration across swallowing trials. Interrater reliability for YPRSRS and PAS ratings ranged from 0.58 to 0.87. Vallecular residue was highest for regular solid, while PAS scores were highest for thin liquid at 20 mL.

**Conclusion:**

In IwIPD, swallowing efficiency was strongly associated with swallowing safety across different bolus consistencies and volumes. EAT‐10 scores showed discriminative performance against FEES findings; however, the EAT‐10 should be interpreted as a patient‐reported screening measure rather than a diagnostic test for physiological swallowing impairment. Comprehensive FEES protocols incorporating a range of bolus consistencies and volumes may support more accurate assessment and individualized dysphagia management in this population.

**WHAT THIS PAPER ADDS:**

*What is already known on this subject*

- Dysphagia is highly prevalent in individuals with Idiopathic Parkinson's Disease (IwIPD) and may involve impairments in both swallowing safety and swallowing efficiency, including pharyngeal residue, penetration, and aspiration. Bolus consistency and volume are known to influence swallowing performance. Patient‐reported outcome measures, such as the Eating Assessment Tool‐10 (EAT‐10), are widely used in dysphagia screening, but they cannot replace instrumental swallowing assessment.

*What this study adds to existing knowledge*

- This study demonstrates a strong relationship between pharyngeal residue severity and penetration–aspiration across different IDDSI‐defined bolus consistencies and volumes in IwIPD. The findings show that larger bolus volumes were associated with greater pharyngeal residue severity and higher PAS scores, while swallowing outcomes varied across consistencies and anatomical residue sites. The study also shows that EAT‐10 scores demonstrated discriminative performance against FEES findings related to pharyngeal residue, penetration, and aspiration, while highlighting the need for cautious interpretation of EAT‐10 cut‐off values in this population.

*What are the potential or actual clinical implications of this study?*

- The findings support the importance of evaluating both swallowing safety and swallowing efficiency during instrumental assessment in IwIPD. The EAT‐10 may help guide referral decisions for further instrumental swallowing assessment, but it should not be interpreted as a diagnostic test for physiological swallowing impairment. FEES protocols incorporating a range of bolus consistencies and volumes may support more comprehensive clinical characterization of dysphagia and individualized management in this population.

## 1 Introduction

Idiopathic Parkinson's Disease (IPD), one of the most common neurological disorders, is characterized by dopaminergic neuron loss in the substantia nigra pars compacta (Simon et al. 2020). Neurodegenerative changes in IPD impair all phases of swallowing, causing dysphagia that may lead to aspiration pneumonia, poor medication intake, malnutrition, dehydration, prolonged hospitalization, and even premature death (Altman et al. 2010). Dysphagia is common in IwIPD, although reported prevalence varies according to the assessment method. Kalf et al. (2012) reported a pooled prevalence of 82% for objectively measured dysphagia, whereas a more recent meta‐analysis estimated an overall pooled prevalence of 50.4% (Aghaz et al. 2024). This variability highlights the importance of accurate swallowing assessment in this population.

Flexible Endoscopic Evaluation of Swallowing (FEES) and videofluoroscopy are considered gold‐standard methods for identifying physiological swallowing abnormalities, including pharyngeal residue, penetration, and aspiration (Langmore and Murray 2012). However, it may not be feasible to perform instrumental assessment for every patient in busy neurology clinics (Bours et al. 2009). Patient‐reported outcome measures, such as the EAT‐10, are useful for identifying individuals who may require further swallowing evaluation; however, they cannot replace instrumental assessment. This distinction is particularly important in IwIPD because the discrepancy between subjective swallowing complaints and objective swallowing impairment is well documented, and some patients may under‐report or lack awareness of dysphagia (Kalf et al. 2012; Rangwala et al. 2025). Therefore, patient‐reported screening tools should be interpreted as indicators of swallowing risk to guide referral for instrumental assessment rather than as direct measures of pharyngeal residue, penetration, or aspiration.

Several studies have examined the ability of the EAT‐10 to identify individuals at risk for pharyngeal residue and aspiration across various diagnostic groups (Arslan et al. 2017, Cheney et al. 2015, Donohue et al. 2022, Erensoy et al. 2024, Florie et al. 2021, Gölaç et al. 2023, Schlickewei et al. 2021, Zuniga et al. 2018). The rationale for examining the EAT‐10 in IwIPD lies in its brevity, widespread use, ease of administration, and potential role in supporting referral decisions when instrumental assessment is not immediately available. Given the known discordance between perceived symptoms and objective swallowing impairment in IwIPD, it is clinically important to determine how EAT‐10 scores correspond to FEES findings across different bolus conditions and to define the limitations of its discriminative performance in this population.

In addition to screening considerations, instrumental assessment allows direct rating of swallowing safety and efficiency. During FEES, the Penetration–Aspiration Scale (PAS) is used to assess penetration and aspiration (Rosenbek et al. 1996), while the Yale Pharyngeal Residue Severity Rating Scale (YPRSRS) is used to evaluate pharyngeal residue (Neubauer et al. 2015). Since pharyngeal residue is thought to be predictive of aspiration, studies involving various diagnostic groups have investigated the relationship between pharyngeal residue and penetration–aspiration (Guijo et al. 2025, Pisegna et al. 2020, Shapira‐Galitz et al. 2019). Nordio et al. (2019) reported a significant association between pharyngeal residue and penetration–aspiration in neurogenic dysphagia, underscoring the need fr detailed evaluation across neurological conditions. Recent work has examined the relationships between respiratory–swallow coordination, pharyngeal residue, penetration, and aspiration in IwIPD (Curtis et al. 2024). However, that study primarily focused on respiratory–swallow coordination as a predictor of swallowing safety and efficiency. The present study extends this work by examining pharyngeal residue and penetration–aspiration across different IDDSI levels and bolus volumes using FEES and by additionally evaluating the discriminative performance of the EAT‐10 against FEES findings.

Standardization remains important in instrumental swallowing assessment (Swan et al. 2019). Since penetration or aspiration may vary between repeated sips of thin liquids or across different volumes and textures, evaluating multiple consistencies and volumes has been emphasized (Steele et al. 2019). Therefore, protocols incorporating diverse consistencies and volumes, together with interrater reliability assessment, may strengthen the reproducibility and clinical interpretability of FEES findings (Gandhi and Steele 2022). The primary aim of this study was to investigate the relationship between pharyngeal residue and penetration–aspiration across different volumes and IDDSI consistencies in IwIPD. The secondary aim was to examine the discriminative performance of the EAT‐10 against FEES findings related to pharyngeal residue, penetration, and aspiration. The tertiary aim was to evaluate interrater reliability for residue and penetration–aspiration ratings across different consistencies and volumes. We hypothesized that greater pharyngeal residue severity would be associated with higher penetration–aspiration scores, and that EAT‐10 scores would demonstrate discriminative performance against FEES findings of pharyngeal residue, penetration, and aspiration, without replacing instrumental assessment.

## 2 Material and Methods

### 2.1 Study Design and Participants

This observational descriptive study was approved by the Health Sciences Research Ethics Committee of Hacettepe University (Research No. GO 22/1251). The sample size was calculated using the G*Power software based on preliminary data obtained from a pilot study involving 16 individuals diagnosed with IPD. With a 95% confidence level (1‐α), 95% statistical power (1‐β), and an effect size of *ρ* = 0.34, the minimum required sample size was determined to be 81 participants. Participants were recruited from individuals referred for swallowing evaluation to the Ondokuz Mayıs University Center for Education, Research, and Practice in Speech and Language Disorders between March 2023 and September 2024. Eligible participants were those diagnosed with IPD by neurologists involved in the study and who met the inclusion criteria.

Inclusion Criteria:

- Age 18 years or older.
- Diagnosed with IPD by a neurologist according to the United Kingdom Parkinson's Disease Society Brain Bank (UK‐PDSBB) criteria and receiving regular antiparkinsonian pharmacological treatment for at least one year to reduce medication‐related variability in swallowing performance.
- Hoehn and Yahr staging performed by a neurologist for individuals diagnosed with IPD.
- Scheduled for a Flexible Endoscopic Evaluation of Swallowing (FEES) following a clinical swallowing assessment.
- FEES performed at least 30 m after medication intake to ensure assessment during a clinically stable medicated state.

Exclusion Criteria:

- Inability to comprehend instructions during the assessment.
- Diagnosis of a condition other than IPD that may cause dysphagia (e.g., head and neck cancer, stroke, amyotrophic lateral sclerosis, chronic heart disease, advanced dementia).
- Prior treatment for dysphagia.
- Presence of another known neurological disorder in addition to IPD.
- Undergoing Deep Brain Stimulation (DBS) as part of IPD treatment.
- Diagnosis of a psychiatric disorder.
- Inability to tolerate FEES during the dysphagia assessment (e.g., nasal bleeding, excessive gagging, vomiting).
- Use of a nasogastric tube.

A total of 138 IwIPD were referred for dysphagia evaluation over 18 months. Forty‐one were excluded due to factors such as cognitive difficulty (*n* = 5), comorbid conditions (*n* = 6), prior dysphagia treatment (*n* = 2), other neurological diagnoses (*n* = 4), DBS surgery (*n* = 4), non‐idiopathic parkinsonism (*n* = 3), psychiatric treatment (*n* = 3), nasogastric tube use (*n* = 4), and inability to tolerate FEES (*n* = 10). Thus, 97 participants were included with written informed consent.

#### 2.1.1 Dysphagia Evaluation

Data collection for the 97 participants who met the inclusion criteria was completed using a detailed case history, clinical swallowing assessment, and FEES. A form was used to document the patient's health information. Additionally, complaints related to swallowing difficulties were examined, including the onset of dysphagia, its progression over time, current status, dietary habits, history of pneumonia, previous swallowing evaluations, and treatments received.

#### 2.1.2 Functional Oral Intake Scale (FOIS)

The FOIS is a 7‐level scale developed to assess current feeding status and any modifications in nutritional intake, particularly in neurogenic populations. Level 7, the highest level, indicates that the individual is able to consume all food and liquid consistencies without restrictions, whereas level 1, the lowest level, indicates complete dependence on tube feeding with no oral intake (Crary et al. 2005).

#### 2.1.3 Eating Assessment Tool‐10 (EAT‐10)

The EAT‐10 evaluates the severity of swallowing difficulties, their impact on quality of life, and the effectiveness of swallowing therapy. The tool is simple and quick to administer, consisting of 10 items, each scored on a scale from 0 to 4 (0 = no problem, 4 = severe problem). A total score of 0 indicates no swallowing difficulty, whereas a maximum score of 40 reflects severe impairment (Demir et al. 2016).

#### 2.1.4 Flexible Endoscopic Evaluation of Swallowing (FEES)

**FIGURE 1:**
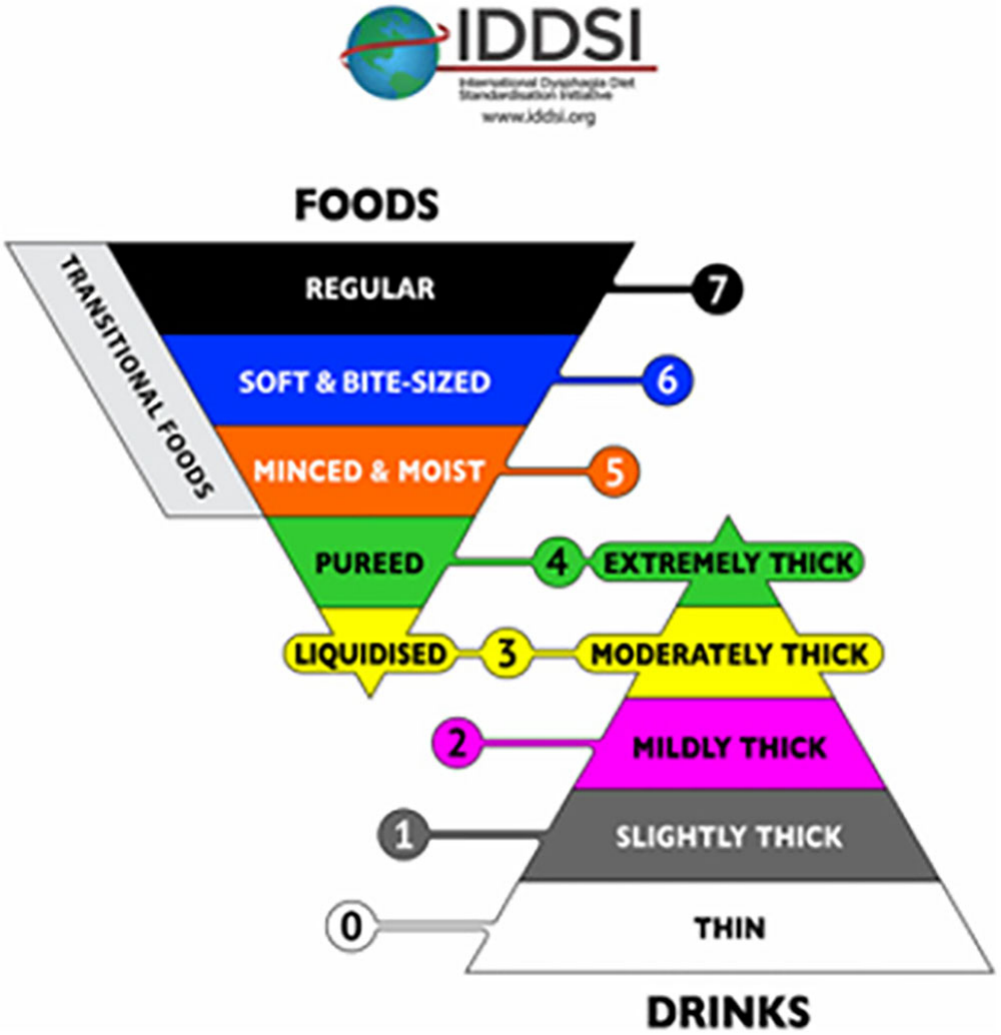
IDDSI Framework (Cichero et al. 2017).

FEES evaluations were made by an otolaryngologist and a Speech Language Therapist (SLT) collaboratively. A flexible nasopharyngoscope (Karl Storz, 3.5 mm diameter, 300 mm length) was used for the FEES procedure. Prior to the examination, the consistency and volume of the test boluses were determined by the SLT in accordance with the IDDSI. In this study, IDDSI levels 0, 1, 2, 4, and 7 were utilized. These levels were selected to represent a clinically feasible range from thin liquids to regular solids while limiting fatigue, examination duration, and potential carryover effects during FEES. IDDSI levels 3, 5, and 6 were not included to maintain a standardized and tolerable protocol across participants. IDDSI, which was developed to globally standardize food and liquid consistencies, defines a 7‐level pyramid to categorize texture and thickness levels (Figure 1) (Cichero et al. 2017).

In this study, for IDDSI level 0, water mixed with food‐grade powdered blue dye was used to provide visual contrast during FEES. Swallowing trials were conducted using one teaspoon (5 mL), one tablespoon (10 mL), and a sequential sip (20 mL). For IDDSI level 1, diluted yogurt drink (*ayran*) was used, and for IDDSI level 2, fermented milk drink (*kefir*) was prepared based on the IDDSI Flow Test results. Liquids prepared at IDDSI levels 1 and 2 were administered in volumes of 5 mL (one teaspoon) and 10 mL (one tablespoon). For IDDSI level 4, yogurt mixed with food‐grade powdered blue dye was used, and boluses of 5 mL and 10 mL were given to participants. For IDDSI level 7, a standardized solid bolus was prepared by cutting a piece of commercially available plain petit beurre biscuit to dimensions of 2.54 cm × 2.54 cm × 0.63 cm.

After determining the bolus consistencies and volumes, participants were informed about the procedure and instructed to sit upright in a comfortable position. They were asked to hold the spoon‐fed bolus in their mouth and swallow upon command. A flexible endoscope was transnasally inserted by the otolaryngologist and positioned to visualize the tongue base, vallecula, larynx, and pyriform sinuses. Each participant completed 10 swallowing trials with the following bolus types: IDDSI level 0 (5, 10, 20 mL), level 1 (5, 10 mL), level 2 (5, 10 mL), level 4 (5, 10 mL), and level 7 (solid). After each swallow, the endoscope was advanced into the laryngeal vestibule to evaluate penetration and aspiration with the PAS and vallecular and pyriform sinus residue with the YPRSRS. Scores were recorded live by the SLT. FEES sessions were video‐recorded from the monitor screen using a Samsung S23 smartphone mounted on a fixed stabilizing holder to enable offline interrater reliability assessment and to prevent data loss in the event of system level recording interruptions. Recordings were transferred to an external drive and deleted from the device. The smartphone recordings were not intended to replace the live clinical FEES assessment. Recordings were checked during offline review, and no screen flicker or artifact interfering with PAS or YPRSRS scoring was observed. For the interrater reliability analysis, FEES recordings from 30 participants were randomly selected using the random selection function in SPSS. This random selection was performed to obtain an unbiased subset of recordings from the full sample for reliability scoring. The same three raters were involved throughout the reliability analysis, consisting of two SLTs and one otolaryngologist. All raters had clinical experience in FEES interpretation and were familiar with PAS and YPRSRS scoring procedures. Before scoring, the raters reviewed the scoring criteria to ensure consistency. They independently reviewed and scored the recordings and were blinded to each other's ratings.

#### 2.1.5 Penetration–Aspiration Scale (PAS)

The PAS is a tool used during instrumental swallowing assessments to help clinicians evaluate airway invasion and its severity while swallowing. The scale ranges from 1 to 8, where 1 indicates that the bolus does not enter the airway, and 8 indicates that the bolus enters the airway, passes below the vocal folds, is not ejected, remains visible, and elicits no response from the patient (Rosenbek et al. 1996).

#### 2.1.6 Yale Pharyngeal Residue Severity Rating Scale (YPRSRS)

The YPRSRS is a five‐point ordinal scale used to assess the severity of pharyngeal residue in the vallecula and pyriform sinuses for each tested consistency, where 1 indicates no residue and 5 indicates severe residue. For the vallecula, the ratings are defined as follows: I–no residue, II–trace coating of the mucosa, III–epiglottic ligament remains visible, IV–epiglottic ligament is obscured, and V–residue fills up to the edge of the epiglottis. For the pyriform sinuses, the scale is defined as: I–no residue, II–trace coating of the mucosa, III–residue fills up to one‐quarter of the wall height, IV–up to half the wall height, and V–residue reaches the aryepiglottic folds (Neubauer et al. 2015).

### 2.2 Statistical Analyses

Data analysis was performed using the Statistical Package for Social Sciences (SPSS), version 26. Descriptive statistics included frequency (n, %), mean, standard deviation, median, and minimum–maximum values. The normality of continuous variables was assessed using Q–Q plots and skewness–kurtosis values. Given the ordinal nature of the YPRSRS and PAS scores and their repeated measurement within the same participants across different bolus consistencies and volumes, comparisons across swallowing conditions were performed using the Friedman repeated‐measures test. These analyses were conducted using complete cases (*n* = 84), as the Friedman test requires complete observations across all repeated swallowing conditions. Kendall's W was calculated as an effect‐size estimate for Friedman analyses. Planned post hoc comparisons were conducted using Wilcoxon signed‐rank tests with Bonferroni correction for six within‐consistency volume comparisons for each outcome. Because repeated swallowing observations were obtained from the same participants, these observations were not treated as independent in comparisons across swallowing conditions. Therefore, within‐participant repeated‐measures analyses were used. PAS and pharyngeal residue scores were considered clinically related but distinct outcomes, reflecting swallowing safety and swallowing efficiency, respectively, and were analysed separately. Spearman's rank correlation coefficients were used to examine the associations between PAS scores, vallecular residue severity, pyriform sinus residue severity, and EAT‐10 scores across swallowing conditions. Additional Spearman correlation analyses were conducted to examine the relationships between disease characteristics, namely Hoehn–Yahr stage and disease duration, and swallowing‐related outcomes, including EAT‐10, FOIS, maximum vallecular residue, maximum pyriform sinus residue, and maximum PAS scores. Reliability analyses were conducted for FEES parameters using recordings from 30 randomly selected participants (31% of the sample). Since interrater agreement was assessed among three raters, Fleiss’ kappa was used to calculate reliability. A kappa value between 0.41–0.60 was interpreted as moderate agreement, 0.61–0.80 as substantial agreement, and 0.81–0.99 as near‐perfect agreement. The discriminative performance of the EAT‐10 against FEES findings related to vallecular residue, pyriform sinus residue, penetration, and aspiration was evaluated using receiver operating characteristic (ROC) curve analysis. The EAT‐10 was treated as a patient‐reported screening measure rather than a diagnostic test. Participants were grouped according to the presence or absence of vallecular residue, pyriform sinus residue, penetration, and aspiration based on FEES findings. The area under the curve (AUC) ranges from 0 to 1, with values closer to 1 indicating higher discriminative ability. The significance level was set at *p* < 0.05.

## 3 Results

The mean age of IwIPD (*n* = 97) was 67.83 ± 6.69 years, while the mean duration since diagnosis was 7.65 ± 3.89 years. Regarding sex distribution, 54 participants (55.7%) were male and 43 (44.3%) were female. Based on disease severity, the distribution across Hoehn and Yahr stages was as follows: Stage 1–27 participants (27.8%), Stage 2–30 participants (30.9%), Stage 3–21 participants (21.6%), Stage 4–13 participants (13.4%), and Stage 5–6 participants (6.2%). The mean EAT‐10 score was 12.47 ± 10.22, the mean FOIS score was 6.15 ± 0.92, and the mean Body Mass Index (BMI) was 23.10 ± 3.31 (Table 1).

**TABLE 1:** Descriptive characteristics of the participants.

	Participants, *n* = 97 (100%)
**Age (years)**	
Mean ± SD	67.83 ± 6.69
Median (min–max)	68 (52–82)
**Duration since IPD diagnosis (years)**	
Mean ± SD	7.65 ± 3.89
Median (min–max)	7 (1–18)
**Sex, *n*(%)**	
Female	43 (44.3)
Male	54 (55.7)
**Hoehn–Yahr stage**	
Mean ± SD	2.39 ± 1.20
Median (min–max)	2 (1–5)
**Hoehn–Yahr stage, *n*(%)**	
Stage 1	27 (27.8)
Stage 2	30 (30.9)
Stage 3	21 (21.6)
Stage 4	13 (13.4)
Stage 5	6 (6.2)
**EAT‐10**	
Mean ± SD	12.47 ± 10.22
Median (min–max)	10 (0–38)
**FOIS**	
Mean ± SD	6.15 ± 0.92
Median (min–max)	6 (4–7)
**BMI (kg/m^2^)**	
Mean ± SD	23.10 ± 3.31
Median (min–max)	22.60 (17.30–35.20)

Abbreviations: BMI: Body Mass Index; EAT–10: Eating Assessment Tool‐10; FOIS: Functional Oral Intake Scale; max: maximum; min: minimum; n: count; SD: standard deviation.

The findings related to pharyngeal residue severity in the vallecula and pyriform sinuses, as well as penetration–aspiration scores across different consistencies and volumes in IwIPD, are presented in Table 2. Repeated‐measures analyses were conducted using complete cases (*n* = 84). Among the 13 participants not included in the complete‐case repeated‐measures analysis, trial non‐completion was primarily due to clinician‐identified safety concerns (*n* = 9) and patient refusal or inability to continue the trial (*n* = 4). Specifically, thin liquid (IDDSI 0) at 20 mL was not completed by 3 participants, one or more slightly thick liquid (IDDSI 1) trials by 5 participants, one or more mildly thick liquid (IDDSI 2) trials by 6 participants, and regular solid (IDDSI 7) trials by 6 participants. These categories overlap because some participants were unable to complete more than one swallowing condition. Friedman tests showed significant differences across swallowing conditions for vallecular residue severity, χ^2^(9) = 272.21, *p* < 0.001, Kendall's W = 0.360; pyriform sinus residue severity, χ^2^(9) = 198.77, *p* < 0.001, Kendall's W = 0.263; and PAS scores, χ^2^(9) = 194.12, *p* < 0.001, Kendall's W = 0.257 (Table 2). The highest mean rank for vallecular residue was observed for regular solid (IDDSI 7), followed by pureed consistency (IDDSI 4) at 10 mL. For pyriform sinus residue, the highest mean rank was observed for mildly thick liquid (IDDSI 2) at 10 mL. For PAS scores, the highest mean rank was observed for thin liquid (IDDSI 0) at 20 mL. Planned post hoc Wilcoxon signed‐rank tests with Bonferroni correction confirmed significant volume‐related differences across all planned within‐consistency comparisons for vallecular residue, pyriform sinus residue, and PAS scores. Table S1 provides the detailed pairwise comparisons, including Z statistics and Bonferroni‐adjusted *p*‐values, for each planned volume comparison.

**TABLE 2:** Comparison of vallecular and pyriform sinus residue severity and penetration–Aspiration scores across different consistencies and volumes.

	Test material	*n*	Mean ± SD	Median (min–max)	Mean rank
**Vallecular residue severity**	Thin liquid (IDDSI 0)–5 mL	84	1.54 ± 0.78	1 (1–4)	3.70
	Thin liquid (IDDSI 0)–10 mL	84	1.70 ± 0.95	1 (1–4)	4.40
	Thin liquid (IDDSI 0)–20 mL	84	2.06 ± 1.18	2 (1–5)	6.01
	Slightly thick liquid (IDDSI 1)–5 mL	84	1.64 ± 0.90	1 (1–4)	4.10
	Slightly thick liquid (IDDSI 1)–10 mL	84	1.92 ± 1.07	2 (1–5)	5.35
	Mildly thick liquid (IDDSI 2)–5 mL	84	1.80 ± 1.05	1 (1–5)	4.78
	Mildly thick liquid (IDDSI 2)–10 mL	84	2.20 ± 1.24	2 (1–5)	6.64
	Pureed (IDDSI 4)–5 mL	84	1.99 ± 1.11	2 (1–5)	5.68
	Pureed (IDDSI 4)–10 mL	84	2.31 ± 1.27	2 (1–5)	7.12
	Regular solid (IDDSI 7)	84	2.36 ± 1.21	2 (1–5)	7.22
	**Overall comparison**	Friedman χ^2^(9) = 272.21; *p* < 0.001; Kendall's W = 0.360			
**Pyriform sinus residue severity**	Thin liquid (IDDSI 0)–5 mL	84	1.58 ± 0.95	1 (1–4)	3.68
	Thin liquid (IDDSI 0)–10 mL	84	1.86 ± 1.16	1 (1–5)	4.74
	Thin liquid (IDDSI 0)–20 mL	84	2.20 ± 1.35	2 (1–5)	6.36
	Slightly thick liquid (IDDSI 1)–5 mL	84	1.80 ± 1.15	1 (1–5)	4.48
	Slightly thick liquid (IDDSI 1)–10 mL	84	2.14 ± 1.26	2 (1–5)	6.10
	Mildly thick liquid (IDDSI 2)–5 mL	84	1.93 ± 1.22	1 (1–5)	5.08
	Mildly thick liquid (IDDSI 2)–10 mL	84	2.30 ± 1.39	2 (1–5)	6.79
	Pureed (IDDSI 4)–5 mL	84	1.89 ± 1.18	1 (1–5)	4.98
	Pureed (IDDSI 4)–10 mL	84	2.25 ± 1.33	2 (1–5)	6.56
	Regular solid (IDDSI 7)	84	2.15 ± 1.19	2 (1–5)	6.23
	**Overall comparison**	Friedman χ^2^(9) = 198.77; *p* < 0.001; Kendall's W = 0.263			
**Penetration–Aspiration Scale score**	Thin liquid (IDDSI 0)–5 mL	84	2.35 ± 1.90	1 (1–7)	5.44
	Thin liquid (IDDSI 0)–10 mL	84	2.67 ± 2.21	1 (1–8)	6.37
	Thin liquid (IDDSI 0)–20 mL	84	3.10 ± 2.39	1 (1–8)	7.63
	Slightly thick liquid (IDDSI 1)–5 mL	84	2.14 ± 1.78	1 (1–7)	4.93
	Slightly thick liquid (IDDSI 1)–10 mL	84	2.46 ± 2.04	1 (1–8)	5.96
	Mildly thick liquid (IDDSI 2)–5 mL	84	1.95 ± 1.60	1 (1–6)	4.36
	Mildly thick liquid (IDDSI 2)–10 mL	84	2.36 ± 1.95	1 (1–7)	5.61
	Pureed (IDDSI 4)–5 mL	84	1.87 ± 1.45	1 (1–6)	4.07
	Pureed (IDDSI 4)–10 mL	84	2.23 ± 1.83	1 (1–7)	5.17
	Regular solid (IDDSI 7)	84	2.29 ± 1.85	1 (1–8)	5.46
	**Overall comparison**	Friedman χ^2^(9) = 194.12; *p* < 0.001; Kendall's W = 0.257			

*Note*: Comparisons across swallowing conditions were performed using the Friedman repeated‐measures test. Higher mean ranks indicate greater residue severity or higher PAS scores.

Abbreviations: IDDSI = International Dysphagia Diet Standardisation Initiative; PAS = Penetration–Aspiration Scale; SD = standard deviation.

The correlation values between PAS scores, YPRSRS scores, and EAT‐10 scores across swallowing conditions are presented in Table 3. PAS scores were positively correlated with vallecular residue severity across all conditions, with correlation coefficients ranging from *ρ* = 0.80 to *ρ* = 0.88, *p* < 0.01. PAS scores were also positively correlated with pyriform sinus residue severity across all conditions, with correlation coefficients ranging from *ρ* = 0.85 to *ρ* = 0.91, *p* < 0.01. Correlations between PAS and EAT‐10 scores were moderate and positive across all swallowing conditions, ranging from *ρ* = 0.56 to *ρ* = 0.64, *p* < 0.01. Correlations between disease characteristics and swallowing‐related outcomes are presented in Table 4. Hoehn–Yahr stage was positively correlated with EAT‐10 scores (*ρ* = 0.629, *p* < 0.01), maximum vallecular residue (*ρ* = 0.371, *p* < 0.01), maximum pyriform sinus residue (*ρ* = 0.377, *p* < 0.01), and maximum PAS scores (*ρ* = 0.355, *p* < 0.01), and negatively correlated with FOIS scores (*ρ* = −0.573, *p* < 0.01). Disease duration was positively correlated with EAT‐10 scores (*ρ* = 0.559, *p* < 0.01), maximum vallecular residue (*ρ* = 0.436, *p* < 0.01), maximum pyriform sinus residue (*ρ* = 0.446, *p* < 0.01), and maximum PAS scores (*ρ* = 0.449, *p* < 0.01), and negatively correlated with FOIS scores (*ρ* = −0.604, *p* < 0.01).

**TABLE 3:** Correlations between PAS scores and YPRSRS, and EAT–10 across different consistencies and volumes.

Test material	Val–residue	Ps–residue	EAT–10
Thin liquid (IDDSI 0)–5 mL	0.81^**^	0.87^**^	0.59^**^
Thin liquid (IDDSI 0)–10 mL	0.83^**^	0.89^**^	0.64^**^
Thin liquid (IDDSI 0)–20 mL	0.88^**^	0.91^**^	0.61^**^
Slightly thick liquid (IDDSI 1)–5 mL	0.84^**^	0.87^**^	0.56^**^
Slightly thick liquid (IDDSI 1)–10 mL	0.87^**^	0.85^**^	0.59^**^
Mildly thick liquid (IDDSI 2)–5 mL	0.83^**^	0.89^**^	0.61^**^
Mildly thick liquid (IDDSI 2)–10 mL	0.86^**^	0.91^**^	0.64^**^
Pureed (IDDSI 4)–5 mL	0.84^**^	0.88^**^	0.62^**^
Pureed (IDDSI 4)–10 mL	0.87^**^	0.88^**^	0.64^**^
Regular solid (IDDSI 7)	0.80^**^	0.86^**^	0.60^**^

*Note*: Values are Spearman's correlation coefficients.Abbreviations: EAT‐10 = Eating Assessment Tool‐10; IDDSI = International Dysphagia Diet Standardisation Initiative; Ps = pyriform sinus residue; Val = vallecular residue.

** *p* < 0.01.

**TABLE 4:** Correlations between disease characteristics and swallowing outcomes.

Outcome	Disease severity (ρ)	Disease duration (ρ)
EAT‐10	0.63^**^	0.56^**^
FOIS	−0.57^**^	−0.60^**^
Maximum vallecular residue	0.37^**^	0.44^**^
Maximum pyriform sinus residue	0.38^**^	0.45^**^
Maximum PAS	0.36^**^	0.45^**^

*Note*: Values are Spearman's rank correlation coefficients (ρ). Hoehn–Yahr stage was used as the indicator of disease severity. Maximum residue and PAS scores represent the highest score observed across all swallowing conditions.

Abbreviations: EAT‐10 = Eating Assessment Tool‐10; FOIS = Functional Oral Intake Scale; PAS = Penetration–Aspiration Scale.

** *p* < 0.01.

**FIGURE 2:**
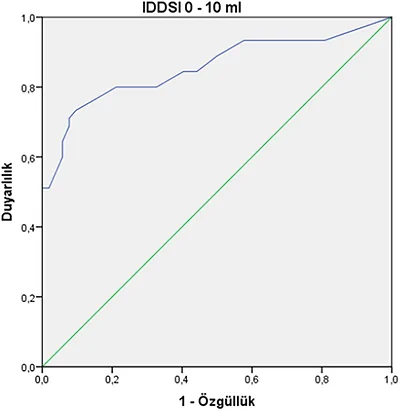
ROC curve showing the discriminative performance of the EAT‐10 for vallecular residue at thin liquid (IDDSI 0)–10 mL.

**FIGURE 3:**
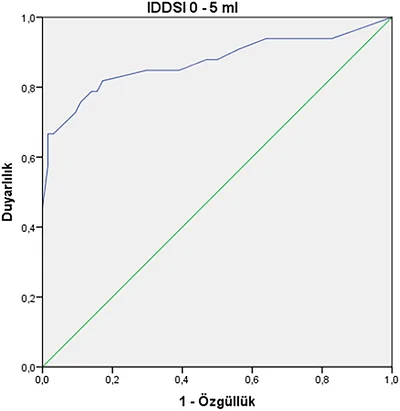
ROC curve showing the discriminative performance of the EAT‐10 for pyriform sinus residue at thin liquid (IDDSI 0)–5 mL.

**FIGURE 4:**
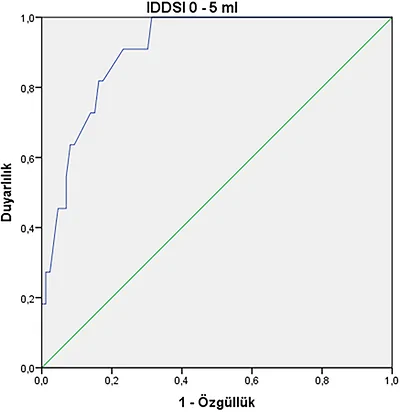
ROC curve showing the discriminative performance of the EAT‐10 for aspiration at thin liquid (IDDSI 0)–5 mL.

**FIGURE 5:**
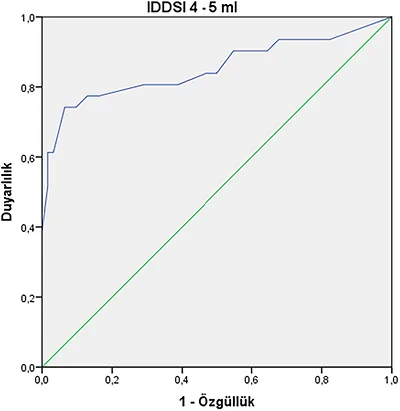
ROC curve showing the discriminative performance of the EAT‐10 for penetration at pureed (IDDSI 4)–5 mL.

The ROC analysis results for the discriminative performance of the EAT‐10 are presented in Table 5. For vallecular residue, AUC values ranged from 0.752 to 0.856 across swallowing conditions. For pyriform sinus residue, AUC values ranged from 0.778 to 0.872. For penetration, AUC values ranged from 0.720 to 0.852. For aspiration, AUC values ranged from 0.810 to 0.908. The highest AUC for aspiration was observed for thin liquid (IDDSI 0) at 5 mL, with a cut‐off score of ≥19, sensitivity of 81.8%, and specificity of 82.6%. Representative ROC curves for the highest AUC values are shown in Figures 2, 3, 4, 5.

**TABLE 5:** ROC analysis results for the discriminative performance of the EAT‐10 for pharyngeal residue, penetration, and aspiration across different bolus consistencies and volumes.

	Test material	AUC (95% CI)	Sens. %	Spec. %	Cut‐off
**Vallecula**	Thin liquid (IDDSI 0)–5 mL	0.851 (0.762–0.941)	81.6	72.9	≥12
	Thin liquid (IDDSI 0)–10 mL	0.856 (0.775–0.937)	80.0	78.8	≥12
	Thin liquid (IDDSI 0)–20 mL	0.844 (0.766–0.922)	75.5	75.6	≥9
	Slightly thick liquid (IDDSI 1)–5 mL	0.848 (0.758–0.938)	79.5	73.6	≥11
	Slightly thick liquid (IDDSI 1)–10 mL	0.818 (0.732–0.904)	71.4	72.1	≥10
	Mildly thick liquid (IDDSI 2)–5 mL	0.833 (0.747–0.919)	75.6	73.9	≥11
	Mildly thick liquid (IDDSI 2)–10 mL	0.815 (0.729–0.901)	73.2	74.3	≥9
	Pureed (IDDSI 4)–5 mL	0.797 (0.710–0.884)	66.7	67.4	≥10
	Pureed (IDDSI 4)–10 mL	0.815 (0.730–0.900)	70.3	75.8	≥9
	Regular solid (IDDSI 7)	0.752 (0.648–0.856)	64.1	70.4	≥9
**Pyriform sinus**	Thin liquid (IDDSI 0)–5 mL	0.872 (0.783–0.961)	81.8	82.8	≥14
	Thin liquid (IDDSI 0)–10 mL	0.840 (0.755–0.925)	77.3	75.5	≥12
	Thin liquid (IDDSI 0)–20 mL	0.802 (0.715–0.890)	69.1	69.2	≥9
	Slightly thick liquid (IDDSI 1)–5 mL	0.847 (0.758–0.936)	78.9	74.1	≥12
	Slightly thick liquid (IDDSI 1)–10 mL	0.788 (0.698–0.879)	69.8	66.7	≥9
	Mildly thick liquid (IDDSI 2)–5 mL	0.827 (0.736–0.918)	76.7	75.0	≥12
	Mildly thick liquid (IDDSI 2)–10 mL	0.801 (0.712–0.899)	71.4	71.4	≥9
	Pureed (IDDSI 4)–5 mL	0.828 (0.742–0.914)	73.9	72.5	≥11
	Pureed (IDDSI 4)–10 mL	0.790 (0.702–0.878)	69.5	68.4	≥9
	Regular solid (IDDSI 7)	0.778 (0.684–0.871)	66.1	64.7	≥9
**Penetration**	Thin liquid (IDDSI 0)–5 mL	0.753 (0.638–0.867)	65.6	66.7	≥10
	Thin liquid (IDDSI 0)–10 mL	0.777 (0.669–0.885)	67.7	66.0	≥9
	Thin liquid (IDDSI 0)–20 mL	0.738 (0.626–0.850)	67.6	61.0	≥8
	Slightly thick liquid (IDDSI 1)–5 mL	0.784 (0.666–0.902)	69.2	70.4	≥11
	Slightly thick liquid (IDDSI 1)–10 mL	0.720 (0.593–0.847)	61.5	62.5	≥9
	Mildly thick liquid (IDDSI 2)–5 mL	0.800 (0.679–0.920)	74.1	69.6	≥11
	Mildly thick liquid (IDDSI 2)–10 mL	0.794 (0.675–0.914)	72.0	72.0	≥12
	Pureed (IDDSI 4)–5 mL	0.852 (0.754–0.951)	80.6	71.0	≥12
	Pureed (IDDSI 4)–10 mL	0.801 (0.680–0.921)	74.1	75.0	≥12
	Regular solid (IDDSI 7)	0.756 (0.641–0.870)	66.7	68.6	≥10
**Aspiration**	Thin liquid (IDDSI 0)–5 mL	0.908 (0.840–0.977)	81.8	82.6	≥19
	Thin liquid (IDDSI 0)–10 mL	0.870 (0.756–0.985)	81.3	79.0	≥18
	Thin liquid (IDDSI 0)–20 mL	0.867 (0.759–0.975)	84.2	77.3	≥15
	Slightly thick liquid (IDDSI 1)–5 mL	0.810 (0.665–0.956)	75.0	75.0	≥17
	Slightly thick liquid (IDDSI 1)–10 mL	0.857 (0.742–0.971)	77.8	79.7	≥17
	Mildly thick liquid (IDDSI 2)–5 mL	0.891 (0.803–0.978)	75.0	79.5	≥19
	Mildly thick liquid (IDDSI 2)–10 mL	0.830 (0.702–0.959)	75.0	74.7	≥17
	Pureed (IDDSI 4)–5 mL	0.875 (0.733–0.999)	75.0	71.0	≥18
	Pureed (IDDSI 4)–10 mL	0.889 (0.798–0.981)	85.7	78.3	≥18
	Regular solid (IDDSI 7)	0.874 (0.769–0.980)	81.8	78.7	≥18

*Note*: All ROC analyses were statistically significant at *p* < 0.01.

Abbreviations: AUC = area under the curve; CI = confidence interval; IDDSI = International Dysphagia Diet Standardisation Initiative; Sens. = sensitivity; Spec. = specificity.

The interrater reliability results for vallecular residue severity, pyriform sinus residue severity, and PAS scores are presented in Table 6. Fleiss’ kappa values for vallecular residue severity ranged from 0.71 to 0.86, indicating substantial to near‐perfect agreement. Fleiss’ kappa values for pyriform sinus residue ranged from 0.58 to 0.75, indicating moderate to substantial agreement. Fleiss’ kappa values for PAS scores ranged from 0.69 to 0.87, indicating substantial to near‐perfect agreement.

**TABLE 6:** Inter‐rater reliability for vallecular and pyriform sinus residue severity and penetration–Aspiration scores across different consistencies and volumes.

	Test material	(κ)	Level of agreement
**Vallecular residue severity**	Thin liquid (IDDSI 0)–5 mL	0.71	Substantial
	Thin liquid (IDDSI 0)–10 mL	0.77	Substantial
	Thin liquid (IDDSI 0)–20 mL	0.82	Near perfect
	Slightly thick liquid (IDDSI 1)–5 mL	0.75	Substantial
	Slightly thick liquid (IDDSI 1)–10 mL	0.79	Substantial
	Mildly thick liquid (IDDSI 2)–5 mL	0.81	Near perfect
	Mildly thick liquid (IDDSI 2)–10 mL	0.83	Near perfect
	Pureed (IDDSI 4)–5 mL	0.80	Near perfect
	Pureed (IDDSI 4)–10 mL	0.85	Near perfect
	Regular solid (IDDSI 7)	0.86	Near perfect
**Pyriform sinus residue severity**	Thin liquid (IDDSI 0)–5 mL	0.58	Moderate
	Thin liquid (IDDSI 0)–10 mL	0.65	Substantial
	Thin liquid (IDDSI 0)–20 mL	0.70	Substantial
	Slightly thick liquid (IDDSI 1)–5 mL	0.68	Substantial
	Slightly thick liquid (IDDSI 1)–10 mL	0.71	Substantial
	Mildly thick liquid (IDDSI 2)–5 mL	0.73	Substantial
	Mildly thick liquid (IDDSI 2)–10 mL	0.75	Substantial
	Pureed (IDDSI 4)–5 mL	0.72	Substantial
	Pureed (IDDSI 4)–10 mL	0.74	Substantial
	Regular solid (IDDSI 7)	0.71	Substantial
**Penetration**–**Aspiration**	Thin liquid (IDDSI 0)–5 mL	0.69	Substantial
	Thin liquid (IDDSI 0)–10 mL	0.74	Substantial
	Thin liquid (IDDSI 0)–20 mL	0.83	Near perfect
	Slightly thick liquid (IDDSI 1)–5 mL	0.73	Substantial
	Slightly thick liquid (IDDSI 1)–10 mL	0.81	Near perfect
	Mildly thick liquid (IDDSI 2)–5 mL	0.82	Near perfect
	Mildly thick liquid (IDDSI 2)–10 mL	0.86	Near perfect
	Pureed (IDDSI 4)–5 mL	0.85	Near perfect
	Pureed (IDDSI 4)–10 mL	0.87	Near perfect
	Regular solid (IDDSI 7)	0.81	Near perfect

Abbreviations: (κ): Fleiss’ kappa value. ; IDDSI: International Dysphagia Diet Standardisation Initiative; ml: milliliter.

## 4 Discussion

A multinational consensus on dysphagia in IPD highlighted unresolved issues in timely and accurate diagnosis, the need for future research on the reliability of clinical methods, and the integration of instrumental evaluations. However, studies employing such instrumental tools in IwIPD remain limited (Cosentino et al. 2022). Although FEES and videofluoroscopy are accepted as gold‐standard instrumental methods, variability in bolus protocols, rating procedures, and interpretation remains an important methodological issue in swallowing research (Swan et al. 2019). Nevertheless, validated standardized frameworks, such as the Modified Barium Swallow Impairment Profile (MBSImP) for videofluoroscopy (Martin‐Harris et al. 2017) and DIGEST‐FEES for FEES (Starmer et al. 2021), have been developed to improve standardization and reproducibility in instrumental swallowing assessment. Outcomes of instrumental methods may still vary depending on factors such as bolus consistency and volume, participant characteristics, and whether verbal instructions are provided prior to swallowing (Steele et al. 2019). Therefore, protocols incorporating diverse consistencies and volumes, together with interrater reliability assessment, may strengthen the reproducibility and clinical interpretability of FEES findings (Gandhi and Steele 2022).

The identification and grading of pharyngeal residue have been central objectives of FEES since its early clinical use. However, only a limited number of studies have examined pharyngeal residue severity in IwIPD (Araújo et al. 2024; Battista et al. 2025). Previous studies have reported residue severity across selected IDDSI levels, but anatomical region‐specific reporting across multiple consistency–volume conditions remains limited. Direct comparison across studies is also limited by differences in bolus protocols, IDDSI levels, and reporting approaches (Araújo et al. 2024; Battista et al. 2025). In this context, the present study adds to the literature by separately evaluating vallecular and pyriform sinus residue severity across ten IDDSI‐defined consistency–volume conditions. The present findings support the importance of considering both consistency and volume when assessing swallowing efficiency and safety in IwIPD. Thicker liquids and solids may require greater oral motor force, and reduced tongue or pharyngeal muscle strength may increase pharyngeal residue with such consistencies (Steele et al. 2015). Physiological differences between the vallecula and pyriform sinuses may also explain why thicker consistencies tend to increase vallecular residue more prominently than pyriform sinus residue (Lee et al. 2012). In addition, prior studies have emphasized bolus volume as an important factor in pharyngeal residue and penetration–aspiration (Steele et al. 2015). Consistent with studies using different bolus volumes in IwIPD, the present findings showed that larger volumes were associated with greater residue severity and higher PAS scores, particularly for thin liquids (Gaeckle et al. 2019; Argolo et al. 2015). The lower PAS scores observed with thicker consistencies are also consistent with previous work suggesting that increased bolus viscosity may provide additional time for airway protection in some individuals (Lam et al. 2007; Steele et al. 2015).

Pharyngeal residue has been associated with penetration–aspiration across several clinical populations (Guijo et al. 2025; Nordio et al. 2019; Sabry and Abou‐Elsaad 2023; Shapira‐Galitz et al. 2019). Previous FEES and videofluoroscopic studies have also examined relationships among swallowing physiology, pharyngeal residue, airway invasion, respiratory–swallow coordination, and MBSImP‐based swallowing impairment in PD (Curtis et al. 2024; Rangwala et al. 2026). The present study adds to this body of work by focusing on FEES‐based vallecular and pyriform sinus residue severity across ten IDDSI‐defined consistency–volume conditions and by examining its association with PAS scores and the discriminative performance of the EAT‐10 in IwIPD. Our findings demonstrated strong positive associations between pharyngeal residue and penetration–aspiration across both anatomical regions. This is consistent with previous work showing associations between residue and airway invasion in dysphagic and neurogenic populations (Guijo et al. 2025; Nordio et al. 2019; Sabry and Abou‐Elsaad 2023; Shapira‐Galitz et al. 2019). Given that vagus nerve involvement is common in IPD, impaired pharyngeal clearance and airway protection may partly explain the strength of these associations (Walter et al. 2018).

The relationship between EAT‐10 scores and FEES findings related to pharyngeal residue, penetration, and aspiration also warrants careful interpretation in IwIPD. A meta‐analysis of the EAT‐10 reported a significant association between EAT‐10 scores and PAS, indicating that higher EAT‐10 scores may reflect greater aspiration risk (Zhang et al. 2023). Similarly, Cheney et al. (2015) noted that EAT‐10 scores may reflect aspiration risk. Arslan et al. (2017) reported that the EAT‐10 was associated with unsafe airway protection in neurological patients, while Gölaç et al. (2023) showed that the EAT‐10 was associated with pharyngeal residue but not aspiration in a mixed‐etiology population. In IwIPD, Schlickewei et al. (2021) reported limited discriminative performance of the EAT‐10 for identifying aspiration and residue across consistencies. In the present study, EAT‐10 scores showed moderate positive correlations with PAS scores and demonstrated discriminative performance against FEES findings related to pharyngeal residue, penetration, and aspiration. However, these findings should not be interpreted as evidence that the EAT‐10 directly detects physiological swallowing impairment. Rather, the EAT‐10 may help identify individuals who require further instrumental swallowing assessment. The relatively high aspiration‐related cut‐off values observed in this study should also be interpreted cautiously. These cut‐offs were derived for FEES‐defined aspiration in IwIPD who were able to comprehend assessment instructions and should not be generalized to dysphagia screening more broadly, for which the originally proposed EAT‐10 cut‐off is substantially lower. This discrepancy suggests that EAT‐10 thresholds may need to be outcome‐specific and population‐specific. Using the original low cut‐off to infer aspiration risk in cognitively intact IwIPD may increase false‐positive referrals, whereas higher cut‐offs may identify individuals with more severe self‐perceived swallowing difficulty. The comparatively stronger discriminative performance observed in the present sample may be partly related to the exclusion of participants with cognitive impairment, which could influence symptom perception and self‐report accuracy. Additionally, item 8 of the EAT‐10 (“I cough when I eat”) had high average scores in our sample, possibly reflecting better self‐awareness of airway invasion‐related symptoms. These differences highlight the importance of considering cognitive status and item‐level responses when interpreting EAT‐10 scores. Nevertheless, these findings should be interpreted in light of the known limitations of patient‐reported outcome measures in PD. Rangwala et al. (2025) emphasized that PROMs may not fully capture physiological swallowing impairments and that patient‐reported symptoms alone may overlook clinically important deficits. Therefore, although the EAT‐10 may support screening and referral decisions, it should not be used as a substitute for instrumental swallowing assessment in IwIPD.

Disease‐related characteristics may also be related to swallowing outcomes in IwIPD. Previous studies have suggested that longer disease duration is associated with increased dysphagia risk in IPD (Claus et al. 2020; Umay et al. 2019). Similarly, higher disease severity, commonly assessed using the Hoehn–Yahr stage, has been reported as an important clinical factor associated with dysphagia in this population (Fukuoka et al. 2019; Polychronis et al. 2019). Consistent with these findings, the present study showed that both longer disease duration and higher Hoehn–Yahr stage were associated with higher EAT‐10 scores, greater vallecular and pyriform sinus residue, higher PAS scores, and lower FOIS scores. These findings suggest that more advanced and longer‐standing IPD may be linked to both greater patient‐reported swallowing difficulty and more severe FEES‐based swallowing impairment.

Interrater reliability findings further support the importance of transparent reporting in FEES‐based research. In the present study, FEES recordings were scored by two experienced speech‐language pathologists and one otolaryngologist, and reliability was calculated for each consistency–volume condition and anatomical region. Previous FEES studies have also used kappa‐based reliability analyses (Erensoy et al. 2024; Labeit et al. 2024). In the present study, agreement was generally stronger for vallecular residue and PAS ratings, while pyriform sinus residue showed relatively lower agreement, particularly for thin liquid at 5 mL. This may reflect the difficulty of consistently judging small amounts of rapidly clearing thin‐liquid residue in the pyriform sinuses during FEES. Overall, these findings suggest that bolus characteristics and anatomical region may influence rating consistency and should be considered when interpreting FEES‐based residue and PAS scores.

### 4.1 Limitations

This study has several limitations that should be considered when interpreting the findings. First, although clinically relevant IDDSI‐defined consistencies were systematically evaluated, not all IDDSI levels and pill swallowing were included in the FEES protocol. IDDSI levels 3, 5, and 6 and pill swallowing were excluded to maintain a clinically tolerable protocol and reduce potential fatigue or carry‐over effects across trials. Therefore, the findings cannot be generalized to these consistencies or to medication swallowing. Second, participants with cognitive impairment were excluded, which may have influenced the observed EAT‐10 performance by limiting the sample to individuals who were more likely to provide reliable symptom reports. Accordingly, the EAT‐10 findings should not be generalized to cognitively impaired individuals with IPD. Third, smartphone recordings were used only for offline interrater reliability assessment and did not replace the live clinical FEES assessment; however, agreement between live FEES ratings and screen‐recorded video ratings was not formally tested. This should be considered when interpreting the reliability findings. Fourth, interrater reliability for pyriform sinus residue was moderate only for the thin liquid (IDDSI 0) 5 mL condition, which may reflect the difficulty of consistently judging small amounts of rapidly clearing residue in the pyriform sinuses during FEES. Therefore, findings for this specific condition should be interpreted with caution. Finally, all FEES assessments were conducted at a single time point; therefore, the present findings do not provide information about longitudinal changes in pharyngeal residue or airway invasion over the course of IPD progression.

## 5 Conclusion

This study provides detailed insights into pharyngeal residue, penetration, and aspiration across various consistencies and volumes in IwIPD. The findings add to the literature by systematically grading vallecular and pyriform sinus residue severity across ten IDDSI‐defined consistency–volume conditions, while also examining its relationship with penetration–aspiration. In addition, EAT‐10 scores showed discriminative performance against FEES findings related to pharyngeal residue, penetration, and aspiration across different swallowing conditions. However, the EAT‐10 should be interpreted as a patient‐reported screening measure rather than a diagnostic test for physiological swallowing impairment, and its cut‐off values should be considered in relation to the target outcome and patient population. These results highlight the importance of evaluating both swallowing safety and efficiency in IwIPD and may support the use of the EAT‐10 to guide referral for instrumental swallowing assessment when interpreted cautiously. This broader focus may assist clinicians in clinical decision‐making and contribute to more comprehensive and individualized dysphagia management.

## Ethics Statement

The study was conducted in accordance with the ethical standards of the relevant national and institutional research committees and with the principles of the Declaration of Helsinki. Ethical approval was obtained from the institutional ethics committee (Approval No: 2023/03‐22).

## Consent

All participants provided informed consent prior to participation.

## Conflicts of Interest

The authors declare no conflicts of interest.

## Supporting information




**Supporting File**: jlcd70335‐sup‐0001‐SuppMat.docx

## Data Availability

The data that support the findings of this study are available from the corresponding author upon reasonable request. The data are not publicly available due to privacy and ethical restrictions.
