# Supplementary material for: Relationship Between EAT‐10 Scores, Pharyngeal Residue Severity, and Penetration–Aspiration in Individuals With Idiopathic Parkinson's Disease: A FEES‐Based Correlation and Discriminative Performance Study
Source: Int J Lang Commun Disord. 2026 Sep 10;61(5):e70335. doi: 10.1111/1460-6984.70335 (PMC13560608; doi:10.1111/1460-6984.70335)
Supplement: Supplementary file 1 [file JLCD-61-0-s001.docx]

**Supplementary Table S1.** Pairwise post hoc comparisons of swallowing conditions using the Wilcoxon signed-rank test

| **Outcome** | **Consistency** | **Comparison** | **Z** | **Adjusted p value** |
| --- | --- | --- | --- | --- |
| **Vallecular residue severity** | Thin liquid (IDDSI 0) | 5 vs 10 mL | -4.243 | <0.001 |
|  |  | 5 vs 20 mL | -6.538 | <0.001 |
|  |  | 10 vs 20 mL | -5.657 | <0.001 |
|  | Slightly thick liquid (IDDSI 1) | 5 vs 10 mL | -4.811 | <0.001 |
|  | Mildly thick liquid (IDDSI 2) | 5 vs 10 mL | -5.925 | <0.001 |
|  | Pureed (IDDSI 4) | 5 vs 10 mL | -5.578 | <0.001 |
| **Pyriform sinus residue severity** | Thin liquid (IDDSI 0) | 5 vs 10 mL | -5.099 | <0.001 |
|  |  | 5 vs 20 mL | -6.952 | <0.001 |
|  |  | 10 vs 20 mL | -5.916 | <0.001 |
|  | Slightly thick liquid (IDDSI 1) | 5 vs 10 mL | -5.657 | <0.001 |
|  | Mildly thick liquid (IDDSI 2) | 5 vs 10 mL | -5.657 | <0.001 |
|  | Pureed (IDDSI 4) | 5 vs 10 mL | -5.488 | <0.001 |
| **Penetration–Aspiration Scale score** | Thin liquid (IDDSI 0) | 5 vs 10 mL | -5.151 | <0.001 |
|  |  | 5 vs 20 mL | -6.461 | <0.001 |
|  |  | 10 vs 20 mL | -5.940 | <0.001 |
|  | Slightly thick liquid (IDDSI 1) | 5 vs 10 mL | -3.287 | 0.001 |
|  | Mildly thick liquid (IDDSI 2) | 5 vs 10 mL | -4.707 | <0.001 |
|  | Pureed (IDDSI 4) | 5 vs 10 mL | -5.444 | <0.001 |

Note. Pairwise comparisons were performed using Wilcoxon signed-rank tests following significant Friedman test results. Bonferroni correction was applied for six planned within-consistency comparisons for each outcome; therefore, the adjusted significance threshold was set at p < 0.0083.
